# Anti-Staphylococcal Activities of *Rosmarinus officinalis* and *Myrtus communis* Essential Oils through ROS-Mediated Oxidative Stress

**DOI:** 10.3390/antibiotics12020266

**Published:** 2023-01-28

**Authors:** Khadijetou Hamoud Bowbe, Karima Bel Hadj Salah, Sarra Moumni, Mada F. Ashkan, Abderrahmen Merghni

**Affiliations:** 1 Laboratory of Transmissible Diseases and Biologically Active Substances LR99ES27, Faculty of Pharmacy, University of Monastir, Monastir 5000, Tunisia; 2 Biological Sciences Department, College of Science and Arts, King Abdulaziz University, Rabigh 21911, Saudi Arabia; 3 Laboratory of Pharmaceutical, Chemical and Pharmacological Drug Development LR12ES09, Faculty of Pharmacy, University of Monastir, Monastir 5000, Tunisia; 4 Laboratory of Antimicrobial Resistance LR99ES09, Faculty of Medicine of Tunis, University of Tunis El Manar, Tunis 1007, Tunisia

**Keywords:** essential oil, *R. officinalis*, *M. communis*, *Staphylococcus aureus*, ant-virulence, oxidative stress, ROS

## Abstract

*Rosmarinus officinalis* and *Myrtus communis* essential oils (EOs) are well-known for their ethno-pharmaceutical properties. In the present study, we have analyzed the chemical composition of both EOs by gas chromatography-mass spectrometry. Then we assessed their antibacterial, antibiofilm, and anti-virulence actions against the opportunistic pathogen *Staphylococcus aureus*. The cytotoxic effect of agents tested against this bacterium was investigated by monitoring reactive oxygen-species (ROS) generation and antioxidant-enzyme (catalase) production. Regarding the antistaphylococcal effects, our results showed antibacterial efficacy of both Eos and their combination, where the minimum inhibitory concentrations ranged between 0.7 and 11.25 mg/mL. A combination of tested agents showed the highest anti-hemolytic and anti-protease effects. Additionally, association between EOs displayed more potency against the development of biofilm performed by *S. aureus,* with percentage of removal reaching 74%. The inhibitory impacts of EOs on *S. aureus* virulence factors were discovered to be concentration-dependent. Furthermore, our results provide insight on the abilities of *R. officinalis* and *M. communis* EOs, as well as their potential in combination, to generate ROS and affect oxidative stress enzyme catalase in *S. aureus*, leading to their antagonistic effect against this pathogen.

## 1. Introduction

*Staphylococcus aureus* is one of the major pathogens credited as being responsible for nosocomial and community acquired infections in humans [1,2]. Colonizing the normal commensal flora of humans and many animals, this opportunistic bacterium is considered the main cause of hospital-acquired infections, generating a variety of symptoms, from mild localized infection to potentially fatal sepsis [3]. *S. aureus* is characterized by a variety of virulence factors, such as secretion of various enzymes and toxins [4], allowing it to avoid the host’s immune defense system and participate in tissue invasion and destruction, along with its ability to form biofilms on medical devices and biomaterials [5].

*S. aureus* is one of the biofilm-producing bacteria with high ability to survive in hostile environments and medical or industrial systems [6]. Inside this biological matrix, bacteria became resistant to different classes of antibiotics and ether-disinfectants, making biofilm-related infections more prone to relapse [7,8]. Across years, synthetic antibiotics are used to overcome *S. aureus* antimicrobial resistance. However, such agents showed high toxicity as well as several side effects in patients [9]. This is one of the factors making antibacterial resistance an increasingly serious threat to global public health. In clinical practice, the ineffectiveness of single-component medications in treating microbiological infections caused by resistant bacteria can be avoided using a combination of two or more antibiotics, which increases their potency and aids in treatment [10,11].

Antibacterial drugs’ principal modes of action include inhibition of cell-wall formation and interference with the ribosome, which cause inhibition of protein synthesis. Antibiotics also alter cell membrane activities, interfere with nucleic acid production (inhibition of DNA gyrase and RNA polymerase), and impede metabolic pathways [12]. Changes in the bacterial genome are responsible for bacterial antibiotic resistance [13]: primarily genetic mutations that modify genomic DNA, result in new resistant strains, as well as transfer of genetic material such as plasmids or mobile genetic components containing antibacterial-resistance genes.

The search for new anti-infection agents from natural resources, with different modes of action and competitive effects, has become a necessity [14]. Numerous lines of research are developing new classes of molecules aimed at new “targets” of action in bacteria, in order to circumvent bacterial resistance mechanisms [15]. For instance, Quorum sensing (QS) has been identified as one of the alternate approaches for combating multidrug-resistant pathogens [16]. Through the QS process, several pathogenic bacteria monitor their own population density and control their virulence factors, antibiotic production, biofilm formation, mobility, and swarming [17].

Essential oils (EOs) from medicinal and aromatic plants are of major interest due to their rich bioactive compounds and potent antimicrobial activity [18]. For instance, *Rosmarinus officinalis* is well known for is medicinal properties against respiratory diseases, headaches, illnesses, and neuropsychiatric disorders [19]. It was largely used for culinary purposes [20] and food preservation [21]. Moreover, *Myrtus communis* is a wild aromatic plant with a wide range of biological properties such as antioxidant, anti-inflammatory, and antimicrobial [22].

Given the large number of chemical compounds present in EOs, there are several targets in the bacterial cell exposed to these molecules [23,24]. Closely related to their chemical composition, numerous mechanisms of action of EOs were documented, including: alteration of the cell wall, degradation of the cytoplasmic membrane and alteration of membrane proteins, leakage of cell contents, coagulation of the cytoplasm, depletion of the proton motility force, and induction of oxidative stress in the bacterial cell [25,26]. It was postulated that EOs induce oxidative stress in treated cells [19]. EOs inhibit bacterial growth by enhancing the production of reactive oxygen species (ROS) in the cells, which consequently inhibit certain essential biological processes [20]. In this context, the present study was undertaken to evaluate the antibacterial properties and mode of action of *R. officinalis* and *M. communis* EOs against the opportunistic pathogen *S. aureus*.

## 2. Results

### 2.1. Chemical Characterization of EOs

The results of chemical analysis of *R. officinalis* and *M. communis* EOs by GC/MS and GC/FID are presented in Table 1.

A total of 56 compounds are identified, representing 96.39% and 96.57% of tested EOs. The major constituent of *R. officinalis* EO was found to be 1,8-cineole with a prevalence of 37.56%. This molecule belongs to the class of monoterpene oxides. The major compound of *M. communis* EO was the α-Pinene, with a percentage of 45.3%. This compound belongs to the class of monoterpene hydrocarbons.

### 2.2. Antibacterial Activity

The antibacterial effects of both EOs and their combination are reported as ‘in vitro’ activity as MIC and MBC and summarized in Table 2.

Both tested agents exerted a bacteriostatic effect against the *S. aureus* strain, with MICs values ranging between 0.7 and 11.25 mg/mL, for *R. officinalis* and *M. communis* respectively (*p* < 0.05). MIC obtained from the combination of EOs was better than that of *R. officinalis* EOs. All tested substances showed bacteriostatic activity against *S. aureus* (MBC/MIC > 4).

### 2.3. Antibiofilm Activity

**Figure 1:**
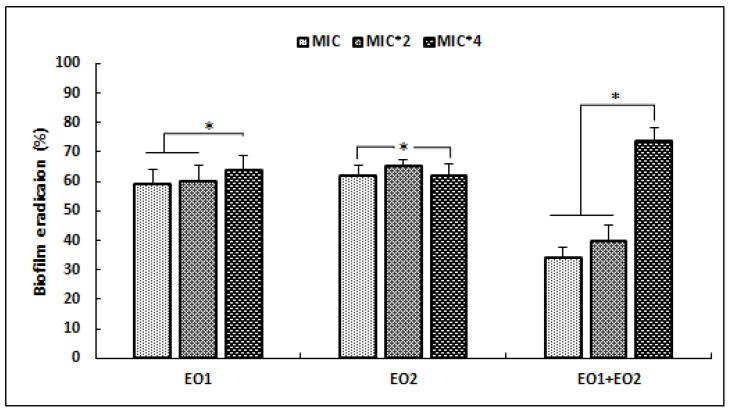
Effects of *R*. *officinalis* (EO1) and *M. communis* (EO2) essential oils and their combination (EO1 + EO2) on the reduction of preformed biofilm of *S. aureus* ATCC 25923, expressed as eradication percentages (%) and evaluated by the Crystal Violet staining assay. Values are the average of at least three independent determinations. Error bars represent standard deviations. (*) Differences were considered significant at *p* < 0.05.

To evaluate the antibiofilm effect of EOs, an established *S*. *aureus* biofilm was treated with various concentrations (MIC, MIC × 2, MIC × 4) of tested agents (Figure 1).

Our results showed that EO1 and EO2 were more effective against the development of preformed biofilm at low concentrations (MIC and 2 × MIC), with percentage reduction values higher than 50%. Whereas, the combination of EOs was more active against *S*. *aureus* biofilm, with percentage reduction value exceeding 74% at high concentration of 4 × MIC (*p* < 0.05). At the same dose (4 × MIC) EO1 and EO2 showed lower antibiofilm activities (*p* < 0.05) that did not exceed 50%.

### 2.4. Antivirulence Activities

The effects of tested EOs against *S. aureus* hemolysin, DNase, and protease production was evaluated by determining the diameter of inhibition halos, in comparison with the untreated strain (Table 3).

Our results showed that the most significant anti-hemolytic effect was obtained with a combination of EOs, resulting in an inhibition diameter equal to 9 ± 0.8 mm after treatment with a concentration of MIC × 4 (*p* < 0.05). At the same concentration, both EO1 and EO2 exhibited lower anti-hemolytic activities (*p* > 0.05).

Regarding the anti-DNase activity, EO of *M. communis* showed more potent effects when compared to other agents without significant difference (*p* > 0.05). Furthermore, tested EOs were found to be effective in the inhibition of *S. aureus* protease activities with a concentration-dependent manner. The most significant anti-protease activity was obtained with the combination of EOs (MIC × 4), with an inhibition diameter reaching 11.6 ± 0.5 mm (*p* < 0.05). At the same concentration, EO1 was found to be less effective against the protease activity of *S. aureus* (*p* < 0.05).

### 2.5. ROS Generation

**Figure 2:**
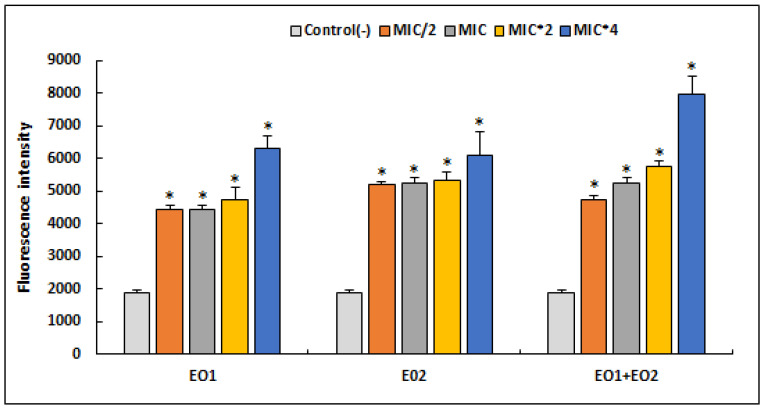
Quantitation of intracellular ROS production by *S. aureus* ATCC 25923 after 24 h treatment with different concentrations of *R*. *officinalis* (EO1) and *M. communis* (EO2) essential oils and their combination (EO1 + EO2), using the DCFA-DA probe. Results are expressed as mean fluorescence intensity ± SD. Asterisks represent significant difference (*p* < 0.05) between each treatment with the negative control.

In this part of our study, we tested the implication of EOs in *S. aureus* oxidative stress through ROS generation (Figure 2).

We have found that tested EOs as well as their association caused an increase in the production of reactive oxygen species compared to the control (*p* < 0.05). It was deduced that equal proportion of *R. officinalis* (50%) and *M. communis* (50%) strongly induced oxidative stress in an *S. aureus* strain treated with a MIC × 4, triggering a high production of ROS in a dose-dependent manner. At the same concentration (MIC × 4), both EO1 and EO2 showed high production of ROS (*p* > 0.05) when compared to their combination (EO1 + EO2).

### 2.6. Catalase Activity

**Figure 3:**
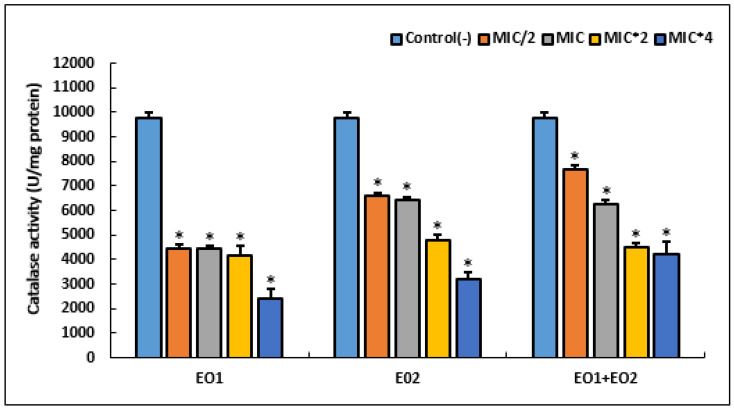
Effect of different concentrations of *R*. *officinalis* (EO1) and *M. communis* (EO2) essential oils and their combination (EO1 + EO2) on the activity of oxidative stress enzyme (catalase) in *S. aureus* ATCC 25923. Bacteria were incubated with various concentrations of EOs for 24 h. An asterisk represents a significant difference (*p* < 0.05) between each treatment and the negative control.

After treatment of an *S. aureus* strain with different concentrations of EOs, we measured the catalase activity, since this enzyme is involved in the defense of the bacterial cell against oxidative stress. Results of this test are presented in Figure 3.

Our results showed that the highest catalase activity (9750 U/mg protein) was observed in an untreated cell. Treatment with various MIC of EOs and their combination revealed a significant decrease in this anti-oxidant activity, in a dose dependent manner (*p* < 0.05). At high concentration (MIC × 4) of tested agents, EO1 showed the lowest catalase activity (*p* < 0.05).

## 3. Discussion

The first part of our investigation was conducted to analyze the chemical composition of *R. officinalis* and *M. communis* EOs. Our results showed that the major constituent of *R. officinalis* EO was found to be 1,8-cineole, which is in agreement with the study of Badreddine et al. [27], having reported a similar value of major compounds of *R. officinalis* EO, which is 1,8-cineole (34.8%). Another recent study conducted by Moumni et al. [28], showed that Tunisian *R. officinalis* EOs are characterized with a high percentage of 1,8-cineole (37.6% to 47.2). Regarding the *M. communis* EO, it was shown that its major compound was found to be the α-Pinene (35.9%) [29], while the study of Cherrat et al. [30] showed the presence of another major compound, Myrtenyl acetate (49.3%). This variability of the chemical composition, even among the same specimens of EOs, is often related to the site of collection as well as geographical provenance. In fact, all these components play a major role in the plant adaptation to the ecology and the environment [31].

In the second part of our investigation, we evaluated the anti-staphylococcal activities of both EOs and their combination. The obtained results showed that all tested substances showed bacteriostatic activity against *S. aureus*. This finding was confirmed by previous studies having shown that EO of *R. officinalis*, with a high amount of 1,8-cineole, excreted a bacteriostatic effect against *S. aureus* strains [32]. Additionally, the same effect was previously reported with *M. communis* EO against *Escherichia coli* and *S. aureus* strains [33]. EOs exert various cytotoxic actions on bacterial strains, acting on several cellular structures. Thus, by crossing the bacterial wall and the plasma membrane, the aromatic compounds of EOs permeabilize the cell membrane and disrupt its function. They can make it permeable to protons and various ions, and inhibit the production of ATP. Eventually, this can lead to lysis of the bacteria. EOs can also reduce membrane fluidity, which impairs the proper functioning of the bacteria [34]. It has been shown that Gram-positive bacteria are more sensitive to EOs than Gram-negative ones. Indeed, it seems that the outer membrane of Gram-negative bacteria, rich in lipopolysaccharides, is more complex and represents an obstacle for aromatic molecules to reach the cytoplasm of this bacterium. However, these active compounds can more easily integrate into the cytoplasm of Gram-positive bacteria given the absence of the complex extra-membrane system, resulting in higher antibacterial activity [35].

Since microbial biofilms increase bacterial resistance to various antimicrobial agents, investigation of antibiofilm effects of bioactive substances from natural resources, such as EOs, remain of interest. Our results revealed that tested EOs and their association displayed high potency against the development of biofilm performed by *S. aureus*. Previously, the effectiveness EOs against performed methicilin-resistant *S*. *aureus* (MRSA) biofilms was reported [36,37]. Generally, the effect of EOs on bacterial biofilm depends on their compositions and on the bacterial strains tested. For example, a previous study reported that *M. communis* EO exhibited significant anti-biofilm activity against *S. aureus* strains, which could be attributed to their α-Pinene richness [38]. Another study conducted by Jardak et al. [39]. showed that Tunisian *R. officinalis* EO exerted a significant *S. epidermidis* biofilm eradication percentage of 67%. Similarly, another study showed that two varieties of Tunisian *Laurus nobilis* EO are capable of inhibiting biofilms of oral *S. aureus* strains, with eradication percentages ranging from 50 to 79% [31]. EOs affect biofilm formation through damage to the outer envelope of this bacterial structure, resulting in the loss of integrity of this layer. Similarly, EOs can also cause inhibition of biofilm synthesis proteins, preventing the development and maturation phase [40]. Within a biofilm, cell-cell interactions and communications have been described. These interactions involve chemical signals, such as quorum sensing (QS) self-inducers [41].

QS is an intercellular communication system that plays an essential role in biofilm formation and virulence-factor production in several bacterial species [17]. Thus, this communication mechanism is used by these microbes to express various survival or virulence traits leading to increased resistance of bacteria [40]. Numerous biosynthetic pathways are regulated by QS, including the production of metabolites [42], biosurfactants [43], and antimicrobials [44]. Interestingly, molecules that interfere with the QS system attenuate bacterial pathogenicity [45]. The activity of tested EOs against *S. aureus* hemolysin production showed that the most significant anti-hemolytic effect was obtained with combination of EOs. Oher findings reported the same hemolytic activity of two EOs of *Dennettia tripetala* on sheep red blood cells [46]. In addition, EOs from *Lippia origanoides* and *Thymus vulgaris* has been demonstrated to have significant effects on hemolytic activity of *S. aureus* ATCC 29213 [47]. Apart their roles of increasing the ability of the infection to establish and remain in humans, various types of *S. arures* hemolysins are produced and associated with a possible activation of QS, prior to biofilm formation [48,49]. Furthermore, our results showed the effectiveness of tested EOs in the inhibition of *S. aureus* DNase and protease activities with a concentration-dependent manner. Therefore, valorization of EOs seems to be an effective strategy to control virulence factors of pathogenic bacteria such as *S. aureus*.

The Implication of EOs in *S. aureus* oxidative stress through ROS generation was investigated. We have found that tested EOs, as well as their association, caused an increase in the production of reactive oxygen species compared to the control, in a dose-dependent manner. Our results are in agreement with a recent study showing that Chamomile EO generated oxidative stress in *S. aureus* ATCC 29213, which may be the main mode of anti-staphylococcal action of this oil [50]. Similarly, oxidative stress was also detected with an increased level of ROS in bacterial cells of *Klebsiella pneumoniae* BAA-1705 and *E. coli* ATCC 25922 treated with Lavender EO [25]. The generation of ROS, including superoxide anions (O^2−^), hydrogen peroxide (H_2_O_2_), and hydroxyl radicals (OH) that are highly reactive, and can lead to oxidative stress if the cell’s antioxidant mechanisms are overcome by pro-oxidant agents [51]. These species are highly reactive, cause oxidative damage and alter the structure and function of macromolecules, such as DNA/RNA, lipids and proteins [52,53]. Even for untreated bacterial cells, ROS production is a natural side effect of aerobic respiration [54], which explains their presence in weak proportions in negative controls.

To protect against the damaging effect of ROS, bacteria are able to produce enzymes such as catalase (CAT) and superoxide dismutase (SOD), to detoxify ROS and accelerate the spontaneous dismutation reaction of H_2_O_2_, along with regulatory mechanisms to counteract their damage [55,56]. After treatment of the *S. aureus* strain with different concentrations of EOs, we measured the catalase activity. Results of this test showed that the highest catalase activity was registered in an untreated cell, while treatment with various MIC of EOs revealed significant decrease in this anti-oxidant activity. In line with our findings, other studies showed decrease in catalase activity in *S. aureus* bacterium exposed to various phyto-compounds such as *Leonurus cardiaca* extract [57], allylpyrocatechol [58], silibin [59], and Catechin [60]. Catalase produced by bacteria facilitates cellular detoxification that allows them to repair or escape oxidative damage from H_2_O_2_ [61]. Reduction of catalase activity caused by biologically active substances might result in increased H_2_O_2_ level and lead to oxidative stress-mediated toxicity in bacterial cells [60]. However, a recent study conducted by Mohammed et al. [62] showed antioxidant capabilities of *Artemisia judaica* EO increasing the content of CAT and SOD enzymes in treated bacteria. It has been reported that a single chemical compound can function as both an antioxidant and a prooxidant [63]. Furthermore, it has been shown in different bacterial species that a short exposure to various antimicrobial agents leads to an increase of catalase enzyme activity in response to this external stress. Whereas, with increasing exposure time (12 to 24 h), bacteria lose their ability to detoxify these antibacterial agents and mitigate the induced stress, leading to a decreases of catalase activity [64,65].

## 4. Materials and Methods

### 4.1. Tested Agents and Bacterial Strain

*Rosmarinus officinalis* and *Myrtus communis* essential oils were purchased commercially from a local producer (KG Flower, Diar ben Salem Béni khiar, Tunisia) after hydrodistillation of fresh aerial parts. For each species, 3 samples of the obtained EO were stored at 4 °C until analysis was attempted. The bacterial strain *Staphylococcus aureus* ATCC 25923 was obtained from the American Type Culture Collection (ATCC). To ensure optimal growth, the bacterial strain was sub-cultured twice, in Brain heart infusion (BHI) broth, and incubated at 37 °C for 24 h before each treatment.

### 4.2. Chemical Characterization of Essential Oils

Quantitative and qualitative analyses of all the chemical composition of studied essential oils were determined by Gas chromatography with flame-ionization detector (GC/FID) and Gas chromatography-mass-spectrometry (GC/MS) as previously described [28].

### 4.3. Antibacterial Activity of EOs and Their Combinations

The minimum inhibitory concentration (MIC) of *R. officinalis* (EO1) and *M. communis* (EO2) EOs against the *S. aureus* strain, as well as their combination (50% EO1 + 50% EO2), was determined by the broth dilution method according to standard protocols [66]. Various concentrations ranging between 0.05 mg/mL and 50 mg/mL of the tested agents were aseptically prepared in 96-well microtiter plates containing Muller Hinton broth (MH) and dimethyl sulfoxide (DMSO). Then inocula (0.5 McFarland) of the tested *S. aureus* strain was added to each well. To determine the minimum bactericidal concentration (MBC) values, MH plates were inoculated with 10 µL from each well medium that had no apparent growth and then incubated for 24 h at 37 °C. MBC was defined as the lowest concentration that killed 99% of the treated bacteria [67].

### 4.4. Antibiofilm Activity

The antibiofilm activity of EO1 and EO2, as well as their combination (EO1 + EO2), were assessed by crystal violet (CV) staining test as described previously [31]. An established *S. aureus* biofilm (48 h) on a sterile 96 microtiter plate was treated with various concentrations of tested agents (1 × MIC, 2 × MIC, and 4 × MIC per well), prepared in DMSO and BHI broth. After incubation for 24 h, the plate was stained with CV (1%) and the biofilm’s biomass was quantified at 570 nm using the microplate reader. The percentage of biofilm eradication was determined by the following formula: [(OD growth control − OD sample)/OD growth control] × 100. Where control is untreated biofilm with EOs or their combination.

### 4.5. Anti-Hemolysin, Anti-DNase and Anti-Protease Activities

The hemolytic activity of the treated *S. aureus* strain was assessed on bacteriological agar supplemented with 5% sheep’s blood for alpha or beta-hemolysin production, and DNase Test Agar Base (DTAB) for the detection of deoxyribonuclease activity [68]. The overnight bacterial culture grown in trypticase soy broth (TSB, Bio-Rad) was diluted (1:100) with the new TSB medium. Then 100 µL of these dilutions were introduced into uniform wells of 6 mm diameter, which were aseptically perforated in the blood and DTAB agar. Following 24 h incubation of each plate at 37 °C, the diameters of the clear zones around the wells were determined [69]. The anti-hemolysin and anti-DNase activities were performed as described below, with the addition of EOs at different concentrations (1 × MIC, 2 × MIC, and 4 × MIC) per well. All the assays were carried out in triplicate.

To check the inhibition of the protein-digesting enzyme protease, the bacterial cells were incubated with varying concentrations (MIC/2; MIC; MIC × 2 and MIC × 4) of EOs and their combination. Then, 10 µL from each treated bacterial cultures were spotted on Bacto agar containing casein (5%), and incubated at 37 °C for 24 h [70]. The cleared zone surrounding the colony was measured and compared to the measured zone obtained from the control (untreated cells).

### 4.6. Reactive Oxygen Species (ROS) Generation

The production of ROS by the *S. aureus* strain exposed to EOs was performed using a peroxynitrite indicator, 20–70-dichlorodihydrofluorescein diacetate (DCFH-DA) (SigmaAldrich, UK), which can detect a broad range of ROS [70]. The adjusted bacterial culture (0.5 McF) was treated with different concentrations of EOs and their combination (corresponding to MIC/2, MIC, 2 × MIC, and 4 × MIC), in presence of DCFH-DA at a final concentration of 5 mM in 0.85% saline, and incubated at 37 °C aerobically for 24 h. Untreated bacterial culture was served as a negative control. The fluorescence emission of DCFH-DA was measured at 525 nm using a Tecan microtiter plate reader with an excitation wavelength of 485 nm [71]. Experiment was carried out in triplicate.

### 4.7. Antioxidant Enzyme Activity

The catalase (CAT) enzyme activity was determined after treatment of overnight *S. aureus* cultures with different concentrations of EOs and their combination (MIC/2, MIC, 2 × MIC, and 4 × MIC). Following incubation for 24 h at 37 °C, the treated bacterial culture was centrifuged at 3000 rpm for 10 min, and the resultant pellet was washed twice with PBS. For enzyme assay, the bacterial extract was prepared by resuspending the pellet in 500 μL of cell lysate buffer (10 Mm Tris-HCl, 1 mMEDTA, 0.1% Triton-X-100 and 150 mM NaCl). After incubation at 37 °C for 1 h, the contents were then centrifuged (3000 rpm for 10 min) and the supernatant was collected for enzyme assay [72]. CAT activity in the bacterial extract was determined according to Acuna et al. [73]. In a quartz cuvette, 780 µL of phosphate buffer (KH_2_PO_2_/K_2_HPO_4_, pH7) were introduced to 200 µL H_2_O_2_ (20 mM), to which 20 µL of bacterial cell lysate was added. Then, optical density of the mixture in each bacterial cell was monitored for 60 s (t = 0 s and = 60 s) at a wavelength of 240 nm. One unit (U) of enzyme activity is defined as the amount of enzyme required to convert 1 µmol of H_2_O_2_ in one second.

### 4.8. Statistical Analysis

All the experiments were carried out in triplicate and the data obtained were presented as means ± standard deviations. Data were further analyzed using the one-way analysis of variance (ANOVA) test to calculate the significance of the results: *p* values less than 0.05 were considered significantly statistically different.

## 5. Conclusions

In the context of fighting against pathogenic bacteria, using biological methods, we investigated through this study the anti-staphylococcal activities of two Eos of *R. officinalis* and *M. communis*. Our results displayed the potent efficacy of both Eos and their combination on *S. aureus*, as a representative of Gram-positive bacteria. Additionally, we highlight the anti-virulence properties of tested agents, due to their active compounds. Of significance, we showed EOs exerting considerable oxidative stress internally within cells, which is coupled with reduction of catalase activity, contributing to their antagonistic effect against *S. aureus.* To overcome chemical degradation and prevent the volatilization of bioactive compounds of tested substances, their encapsulation in nanometric systems could offer a promising intervention.

## Figures and Tables

**Table 1:** Chemical composition (%) of *R*. *officinalis* and *M. communis* essential oils.

N °	KI *	Component	*R. officinalis*	*M. communis*
1	923	Tricyclene	0.24	0.00
2	927	α-Thujene	0.11	0.51
3	934	α-Pinene	3.39	45.3
4	949	Camphene	6.52	0.04
5	974	Sabinene	0.05	4.42
6	978	β-Pinene	3.59	0.00
7	992	β-Myrcene	0.05	0.30
8	1002	α-Phellandrene	1.84	0.00
9	1004	δ-2-Carene	0.00	0.72
10	1006	δ-3-Carene	0.24	0.67
11	1012	α-Terpinene	0.40	0.45
12	1025	p-Cymene	0.95	1.64
13	1029	Limonene	2.52	8.45
14	1038	(Z)-β-Ocimene	0.08	0.06
15	1048	(E)-β -Ocimene	0.07	0.23
16	1058	ϒ-Terpinene	0.47	0.39
17	1090	α-Terpinolene	0.35	0.42
18	1032	1.8-Cineole	37.56	22.02
19	1101	Linalool	0.51	2.63
20	1109	α-Thujone	0.07	0.00
21	1119	β-Thujone	0.05	0.00
22	1127	allo-Ocimene	0.00	0.00
23	1127	α-Campholenal	0.04	0.01
24	1139	(E)-pinocarveol	0.00	0.13
25	1146	Camphor	7.10	0.23
26	1158	Isoborneol	0.01	0.00
27	1166	β-Fenchyl alcohol	0.00	0.06
28	1167	Borneol	4.37	0.00
29	1178	Terpinen-4-ol	0.53	0.85
30	1186	(Z)-Pinocarveol	0.00	0.07
31	1186	p-Cymen-8-ol	0.04	0.00
32	1192	α-Terpineol	1.57	1.60
33	1207	Verbenone	0.02	0.00
34	1258	Linalyl acetate	0.00	0.95
35	1287	Bornyl acetate	0.24	0.17
36	1294	Thymol	0.03	0.00
37	1304	Carvacrol	0.00	0.05
38	1325	Myrtenyl acetate	0.00	0.06
39	1350	α-Terpinyl-acetate	0.00	0.08
40	1373	α-Ylangene	0.11	0.00
41	1377	(E)-Methyl cinnamate	0.00	0.07
42	1385	Geranyl acetate	0.36	0.00
43	1420	(E)-Caryophyllene	5.04	0.67
44	1429	Aromadendrene	0.05	0.00
45	1438	α-Guaiene	0.00	0.11
46	1455	α-Humulene	0.75	0.15
47	1462	allo-Aromadendrene	0.00	0.00
48	1496	α-Muurolene	0.27	0.00
49	1498	(E)-β-Guaiene	0.00	0.06
50	1500	α-Amorphene	0.12	0.00
51	1510	δ-Amorphene	0.00	0.06
52	1523	(Z)-Calamenene	0.00	0.13
53	1525	δ-Cadinene	0.65	0.00
54	1584	Caryophyllene oxide	0.06	0.12
55	1593	Vidiflorol	0.03	0.00
56	1656	α-Cadinol	0.00	0.03
		**Total (%)**	96.39	96.57

**N °**: Component number / KI *: Kovats retention index.

**Table 2:** Antibacterial activity of *R*. *officinalis* (EO1) and *M. communis* (EO2) essential oils and their combination (EO1 + EO2) against *S. aureus* ATCC 25923.

EOs	Concentration (mg/mL)	
EO1	MIC	11.25
	MBC	360
	MBC/MIC	32
EO2	MIC	0.7
	MBC	22.5
	MBC/MIC	32.14
EO1 + EO2	MIC	5.63
	MBC	180
	MBC/MIC	31.97

MIC: Minimum Inhibitory Concentration; MBC: Minimum Bactericidal Concentration. MBC/MIC: Ratio of Minimum Inhibitory Concentration/Minimum Bactericidal Concentration.

**Table 3:** Anti-virulence activities of *R*. *officinalis* (EO1) and *M. communis* (EO2) essential oils and their combination (EO1 + EO2) against *S. aureus* ATCC 25923.

	EO1			EO2			EO1 + EO2		
	A-H	A-D	A-P	A-H	A-D	A-P	A-H	A-D	A-P
Control	20 ± 0.0	20 ± 0.0	18.4 ± 0.5	20 ± 0.0	20 ± 0.0	18.4 ± 0.5	20 ± 0.0	20 ± 0.0	18.4 ± 0.5
MIC/2	15.5 ± 0.5 *	18 ± 0.0	16 ± 0.47	14.6 ± 0.5 *	17 ± 0.8	14.6 ± 0.4	13 ± 0.8 *	17 ± 1.4	14.6 ± 0.5
MIC	15.3 ± 0.5 *	17 ± 0.0	16 ± 1.25	14 ± 0.8 *	16.3 ± 1.2	14.5 ± 0.4	12.5 ± 0.5 *	17 ± 0.8	14.3 ± 0.5
MIC × 2	13.6 ± 1.2 *	17 ± 0.0	16 ± 0.47	13.6 ± 0.9 *	16 ± 1.0	13.3 ± 0.9	11 ± 0.8 *	17 ± 0.8	14 ± 0.0
MIC × 4	13 ± 00 *	15.5 ± 0.5 *	16 ± 0	12.6 ± 0.5 *	15.3 ± 0.5 *	13 ± 0.4 *	9 ± 0.8 *	16 ± 0.0	11.6 ± 0.5 *

A-H: Anti-Hemolysin; A-D: Anti-DNase; A-P: Anti-Protease; Control: untreated bacteria; MIC: Minimum Inhibitory Concentration; *: Significant difference (*p* < 0.05) compared to the control.

## Data Availability

Not applicable.
